# Enterohemorrhagic *Escherichia coli* as Causes of Hemolytic Uremic Syndrome in the Czech Republic

**DOI:** 10.1371/journal.pone.0073927

**Published:** 2013-09-06

**Authors:** Monika Marejková, Květa Bláhová, Jan Janda, Angelika Fruth, Petr Petráš

**Affiliations:** 1 National Reference Laboratory for *E. coli* and Shigella, National Institute of Public Health, Prague, Czech Republic; 2 The 3^rd^ Medical Faculty, Charles University Prague, Prague, Czech Republic; 3 Department of Pediatrics, 2^nd^ Medical Faculty, Charles University Prague and the University Hospital Motol, Prague, Czech Republic; 4 National Reference Center for Salmonella and Other Enteric Pathogens, Robert Koch Institute, Wernigerode, Germany; The University of Hong Kong, China

## Abstract

**Background:**

Enterohemorrhagic *Escherichia coli* (EHEC) cause diarrhea-associated hemolytic uremic syndrome (D+ HUS) worldwide, but no systematic study of EHEC as the causative agents of HUS was performed in the Czech Republic. We analyzed stools of all patients with D+ HUS in the Czech Republic between 1998 and 2012 for evidence of EHEC infection. We determined virulence profiles, phenotypes, antimicrobial susceptibilities and phylogeny of the EHEC isolates.

**Methodology/Principal Findings:**

Virulence loci were identified using PCR, phenotypes and antimicrobial susceptibilities were determined using standard procedures, and phylogeny was assessed using multilocus sequence typing. During the 15-year period, EHEC were isolated from stools of 39 (69.4%) of 56 patients. The strains belonged to serotypes [*fliC* types] O157:H7/NM[*fliC*_H7_] (50% of which were sorbitol-fermenting; SF), O26:H11/NM[*fliC*_H11_], O55:NM[*fliC*_H7_], O111:NM[*fliC*_H8_], O145:H28[*fliC*_H28_], O172:NM[*fliC*_H25_], and Orough:NM[*fliC*_H25_]. O26:H11/NM[*fliC*_H11_] was the most common serotype associated with HUS (41% isolates). Five *stx* genotypes were identified, the most frequent being *stx*_2a_ (71.1% isolates). Most strains contained EHEC-*hlyA* encoding EHEC hemolysin, and a subset (all SF O157:NM and one O157:H7) harbored *cdt-V* encoding cytolethal distending toxin. *espP*α encoding serine protease EspPα was found in EHEC O157:H7, O26:H11/NM, and O145:H28, whereas O172:NM and Orough:NM strains contained *espP*γ. All isolates contained *eae* encoding adhesin intimin, which belonged to subtypes β (O26), γ (O55, O145, O157), γ2/θ (O111), and ε (O172, Orough). Loci encoding other adhesins (*efa1, lpfA*_O26_, *lpfA*_O157OI-141_, *lpfA*_O157OI-154_, *iha*) were usually associated with particular serotypes. Phylogenetic analysis demonstrated nine sequence types (STs) which correlated with serotypes. Of these, two STs (ST660 and ST1595) were not found in HUS-associated EHEC before.

**Conclusions/Significance:**

EHEC strains, including O157:H7 and non-O157:H7, are frequent causes of D+ HUS in the Czech Republic. Identification of unusual EHEC serotypes/STs causing HUS calls for establishment of an European collection of HUS-associated EHEC, enabling to study properties and evolution of these important pathogens.

## Introduction

Enterohemorrhagic *Escherichia coli* (EHEC) are the pathogenic subgroup of Shiga toxin (Stx)-producing *E. coli* strains that cause human diseases including diarrhea, bloody diarrhea and hemolytic uremic syndrome (HUS). HUS is a severe, potentialy life-threatening condition characterized by non-immune hemolytic anemia, thrombocytopenia, and acute renal failure [1]. HUS caused by EHEC strains is typically preceded with diarrhea (and therefore designated D+ HUS), that usually begins as non-bloody and progresses to bloody after several days [1]. D+ HUS usually affects children under 5 years [1] and is the most common cause of acute renal insufficiency in children. It develops in 10–15% children infected with EHEC O157:H7 [1] and also complicates infections with other EHEC serotypes [2]–[9]. In addition to the kidneys, other organs can be affected during HUS including the central nervous system, the pancreas, the heart, the liver and the lungs [1]. The involvement of the central nervous system is the most severe and is associated with higher mortality [6]. The mortality of D+ HUS during the acute phase is <5%, and there is a high frequency of late renal or non-renal sequelae in survivors [10], [11].

Microvascular endothelial damage is the major pathological change underlying HUS [1], [12]. Stxs are presently the best characterised EHEC virulence factors that cause the microvascular endothelial injury [12]. Stxs are released by EHEC in the intestine, absorbed across the intestinal epithelium into the circulation, and transported to microcapillaries of the target organs, mainly the kidneys and the brain. Here Stxs bind to glycosphingolipids of the globo-series, which are abundantly expressed on both glomerular and brain microvascular endothelial cells [13], [14]. This triggers a complex cascade of events resulting in a multi-organ thrombotic process [12]. Although the Stx family is highly heterogeneous, not all Stx types have been associated with HUS [15], [16]. Stx2 is the most common Stx type found in EHEC isolated from HUS patients [3], [15]–[17], but Stx2c, Stx2d and Stx1 also occur in HUS-associated EHEC [3], [15]. In contrast, Stx2b, Stx2e, Stx2f and Stx2g have not been found at all or are found very rarely among HUS isolates [15], [16], [18]–[20]. A possible explanation for the epidemiological association of Stx2 with severe outcome of EHEC infections is the *in vitro* observation that Stx2 is significantly more cytotoxic towards human renal [21] and human brain [22] microvascular endothelial cells than Stx1.

The classical non-sorbitol-fermenting (NSF) EHEC O157:H7 is the predominant cause of HUS worldwide [1], [23], [24]. In addition, several other EHEC serotypes, the most frequent of which are O26:H11/NM (non-motile), O91:H21, O103:H2/NM, O111:H8/NM, O113:H21, and O145:H25/H28/NM, have been increasingly recognized as causes of HUS in Europe [3]–[5], [7], [17], [25]–[27], North America [8], South America [20], and Australia [28]. EHEC strains that have the capability of causing HUS have been designated HUSEC (HUS-associated *E. coli*) [3].

The role of EHEC as causes of HUS in the Czech Republic has not yet been systematically studied. Here, we investigated stool samples from all patients with D+ HUS hospitalized in pediatric centers in the Czech Republic between 1998 and 2012 for EHEC infection. We determined serotypes of the EHEC isolates, a subset of molecular and phenotypic characteristics, and their phylogenetic relationships. Moreover, we determined susceptibilities of the strains to a spectrum of antimicrobials.

## Materials and Methods

### Ethics Statement

This study was approved by the Ethical Committee of the University Hospital Motol, Prague, Czech Republic. Written informed consent for enrollment in the study and publication of patientś data was obtained from parents of all patients.

### Patients and Stool Samples

During January 1998 to December 2012, stool samples from 56 patients with HUS were investigated for EHEC infection in the Reference Laboratory for *E. coli* and Shigella of the National Institute of Public Health in Prague, Czech Republic. This laboratory is specialized for diagnosis of EHEC infections and receives stools from all patients with HUS from this country. The patients originated from different regions of the Czech Republic and there were no apparent temporal or geographical linkages between them. Thirty-four of 56 patients (60.7%) were boys and 22 (39.3%) were girls. All patients were children (median age, 27.5 months; range, 10 to 85 months). Stool samples were collected between 2 and 15 days (median, 6 days) after the onset of prodromal diarrhea.

### Case Definition

HUS was defined as a case of microangiopathic hemolytic anemia (hematocrit <30% with peripheral evidence of intravascular hemolysis), thrombocytopenia (platelet count <150,000 platelets/mm^3^), and renal insufficiency (serum creatinine concentration higher than the upper limit of the normal range for age) [1].

### Detection of EHEC in and Isolation from Patients Stools

The stool samples were enriched in G.N. Enrichment Broth (Hajna) (Laboratorios Conda, Madrid, Spain) with novobiocin supplement (Oxoid, Hampshire, UK) for 5–7 hours (37°C, 180 rpm) and the enriched cultures were inoculated on Columbia blood agar, sorbitol MacConkey agar (SMAC), cefixime-tellurite (CT)-SMAC agar and enterohemolysin agar (all from Oxoid). Since 2008, the enrichment cultures were additionally examined for the presence of *E. coli* O157, O26, O103, O111, and O145 using an immunomagnetic separation (Dynabeads anti-*E. coli* O157, Dynabeads EPEC/VTEC O26, Dynabeads EPEC/VTEC O103, Dynabeads EPEC/VTEC O111, Dynabeads EPEC/VTEC O145) according to the manufactureŕs (Dynal, Oslo, Norway) protocol and subsequently plated on the above media. After overnight incubation at 37°C, bacterial growth from all four plates was collected in a tube containing 1ml of 0.85% NaCl; 100 µl of this suspension was dilluted 1∶10 in sterile distilled water, heated for 10 min at 100°C and centrifuged (12000 rpm, 10 min). Supernatant was used as a template for PCR with primers KS7-KS8, LP43-LP44, and SK1-SK2 which target *stx*_1a_, *stx*_2a_ and *eae,* respectively [3], [15]. All *stx*-positive samples were further PCR tested for genes of O antigen biosynthetic clusters of *E. coli* O157, O26, O55, O111, O103, O145, O91 and O113 [29]–[31]. To isolate strains from samples with positive *stx* PCR results, bacterial suspensions were restreaked on SMAC, CT-SMAC and enterohemolysin agar plates. *E. coli* O157:H7 was isolated from SMAC and/or CT-SMAC using agglutination of sorbitol-negative colonies in O157 antiserum (Denka Seiken Ltd., Tokyo, Japan). EHEC of the major non-O157 serogroups (O26, O111, O145) were isolated from enterohemolysin agar based on their typical enterohemolytic phenotype combined with agglutination in antisera against the respective *E. coli* O antigens (Sifin, Berlin, Germany; Denka Seiken). If no sorbitol-negative or enterohemolytic colonies were present on SMAC/CT-SMAC or enterohemolysin agar, respectively, multiple colonies from these plates (altogether up to 50) were tested for *stx*_1a_ and *stx*_2a_ genes using PCRs described above. The isolates were confirmed as *E. coli* biochemically (API 20E; bioMérieux, Marcy l’Etoile, France) and using a MALDI-TOF mass spectrometer (Microflex LT, Bruker Daltonics, Germany). Mass spectra were processed using the BioTyper software with the version 3.2.1.0. database. Motility of the isolates was determined directly after isolation as follows: The strains were inoculated into the middle of tubes containing soft (0.5%) agar, incubated at 37°C and observed for growth daily. An isolate was considered motile if it spread out of the original inoculation site during 10 days. If there was no growth from the inoculation site during this time, the isolate was considered nonmotile (NM).

### Detection of other Enteric Bacterial Pathogens in Stools

The presence of *Salmonella* spp., *Shigella* spp., *Yersinia enterocolitica*, and *Campylobacter jejuni* in stools was sought using standard microbiological procedures.

### Serotyping

*stx* positive colonies were serotyped using agglutination in polyvalent and monovalent *E. coli* O antisera (Denka Seiken Co., Ltd., Tokyo, Japan; Sifin, Berlin, Germany; Robert Koch Institute, Wernigerode, Germany) and H antisera (Denka Seiken). The presence of the O26, O55, O111, O145, O157, and O172 antigens was confirmed using PCRs targeting genes of the respective O antigen biosynthetic clusters [29]–[32]. The flagellin subunit–encoding *fliC* gene was subtyped using HhaI restriction fragment length polymorphism as described previously [7].

### Genotypic Characteristics

PCRs were performed in a MyCycler Thermal Cycler (Bio-Rad, München, Germany) using reagents from Top-Bio (Prague, Czech Republic) and primers from Generi Biotech (Hradec Kralove, Czech Republic). All isolates were tested for *stx*_1a_, *stx*_2a_, and their subtypes (*stx*_1c_, *stx*_2b_, *stx*_2c_, *stx*_2d_ *stx*_2e_, *stx*_2f_) using published PCR protocols [15], [16]. Genes encoding other toxins (*cdt-*V, EHEC-*hlyA*, *α-hlyA*), the EHEC serine protease EspP (*espP*), and adhesins (*eae, efa1, lpfA*_O26_, *lpfA*_O157OI-141_, *lpfA*_O157OI-154_, *iha*) were PCR detected as described previously [15], [33]–[38]. *eae* genes were subtyped according to Zhang et al. [39] (*eae* β, γ, ε) and Blanco et al. [40] (*eae* γ2/θ). *espP* genes were subtyped according to Brockmeyer et al. [36]. *terE* and *ureD* used as markers for the *ter* and *ure* gene clusters, which encode tellurite resistance urease production, respectively, were amplified as described previously [41], [42]. Genes within the O island (OI) 122 of EHEC O157:H7 strain EDL933 (*efa1*, *sen*, *nleE*, *nleB*, *pagC*) were detected using published PCR protocols [25], [43].

### Phenotypic Characteristics

Utilization of sorbitol was tested on SMAC. Moreover, utilization of sorbitol, rhamnose and production of lysine decarboxylase (LDC) was evaluated according to the API 20E test kit (bioMérieux). Production of β-D-glucuronidase was investigated using COLItest (Erba Lachema, Brno, Czech Republic). Production of EHEC hemolysin and α hemolysin was sought using enterohemolysin agar and Columbia blood agar (Oxoid), respectively. Production of Stx was tested using a Vero cell cytotoxicity assay [2]. The Stx titer was defined as the highest dilution of culture supernatant that caused cytotoxicity in 50% cells after 3 days of incubation. The Stx types (Stx1, Stx2) were identified using VTEC-RPLA assay (Denka Seiken) according to the manufacturer’s instructions. Resistance to tellurite was determined based on the ability of the strains to grow on CT-SMAC. Urease production was determined using the API 20E test.

### Antimicrobial Susceptibility Testing

Susceptibility to ampicillin, cefotaxime, ceftazidime, gentamicin, trimethoprim/sulfamethoxazole, ciprofloxacin, amikacin, meropenem, piperacillin/tazobactam, tigecycline, chloramphenicol, and nitrofurantoin was tested using the disk diffusion method according to EUCAST breakpoints [44] and standard recommendations [45].

### Multilocus Sequence Typing (MLST)

MLST was performed by sequencing internal fragments of seven housekeeping genes (*adk*, *fumC*, *gyrB*, *icd*, *mdh*, *purA*, and *recA*) as described previously [3]. Sequences were analyzed and the minimum-spanning tree was constructed using the SeqSphere software version 0.9 beta.1 (Ridom GmbH, Münster, Germany). All alleles and sequence types (ST) were assigned in accordance with the MLST website (http://mlst.ucc.ie/mlst/dbs/Ecoli).

## Results

### Clinical Features

Among the 56 patients investigated, 52 (92.9%) had prodromal diarrhea, which was bloody in 31 (59.6%). HUS was diagnosed between 2 and 15 days (median, 6 days) after the onset of diarrhea. The median length of hospitalization was 17 days (range, 4 to 55 days). Three patients (5.4%) died during acute phase of HUS. The causes of the deaths were neurological complications (cerebral edema) in two patients (girls, 18 and 25 months old, both infected with EHEC O26:H11), one of whom also developed lung edema; one patient (boy, 30 months old, infected with sorbitol-fermenting (SF) EHEC O157:NM) died of acute renal failure. Deaths occurred between 3 and 6 days (median, 3 days) after HUS development.

### Serotypes and *fliC* Genotypes of EHEC Isolates

Stool samples from 39 of 56 patients (69.6%) were positive in screening for *stx*_1a_ and/or *stx*_2a_ genes using PCR. EHEC strains were isolated from all of these 39 *stx*-positive stool samples. Thirty-seven isolates belonged to eight different serotypes and two were non-typeable (Orough) (Table 1). The most common serotype was O26:H11/NM (non-motile), which accounted for 16 (41%) of 39 isolates (Table 1). The second most common serotype was O111:NM (six of 39 isolates; 15.4%). NSF EHEC O157:H7/NM were isolated from five (12.8%) patients and an additional five patients (12.8%) were positive for SF EHEC O157:NM strains (Table 1). One isolate belonged to serotype O172:NM, which has been rarely isolated from patients with HUS [25].

**Table 1:** Serotypes and genotypic characteristics of EHEC strains isolated from patients with HUS in the Czech Republic, 1998–2012.

Virulence locus^a^	Serotype (number of strains)^b^							
	O157:H7/NM (NSF)	O157:NM (SF)	O55:NM	O26:H11/NM	O111:NM	O145:H28	O172:NM	Orough:NM
	*fliC*_H7_ (n = 5)	*fliC*_H7_ (n = 5)	*fliC*_H7_ (n = 2)	*fliC*_H11_ (n = 16)	*fliC*_H8_ (n = 6)	*fliC*_H28_ (n = 2)	*fliC*_H25_ (n = 1)	*fliC*_H25_ (n = 2)
*stx*_1a_	−c	–	–	+ (1)^c^	+ (2)	–	–	–
*stx*_2a_	+ (1)	+ (4)^d^	+	+ (15)	–	+	+	+
*stx*_1a_ *+stx*_2a_	–	–	–	–	+ (4)	–	–	–
*stx*_1a_ *+stx*_2c_	+ (2)	–	–	–	–	–	–	–
*stx*_2a_ *+stx*_2c_	+ (2)	–	–	–	–	–	–	–
*cdt*–*V*	+ (1)	+	–	–	–	–	–	–
EHEC–*hlyA*	+	+ (4)	–	+	+ (4)	+	+	+
α–*hlyA*	–	–	–	–	–	–	–	–
*espP* e	+ (α)	–	–	+ (α) (9)	–	+ (α) (1)	+ (γ)	+ (γ)
*eae* f	+ (γ)	+ (γ)	+ (γ)	+ (ß)	+ (γ2/θ)	+ (γ)	+ (ε)	+ (ε)
*efa1*	+^g^	+	+	+	+	+^g^	+	+
*lpfA*_O26_	–	–	–	+	+	–	–	–
*lpfA*_O157/OI_–_141_	+	+	+	–	–	+	–	–
*lpfA*_O157/OI_–_154_	+	+	+	–	–	–	–	–
*iha*	+	–	–	+	+ (5)	+	–	–
*terE*	+	–	–	+	+ (5)	+	–	–
*ureD*	+	–	–	+	+ (5)	+	–	–
*irp2*	–	–	–	+	–	–	–	–
*fyuA*	–	–	–	+	–	–	–	–

a The genes encode the following proteins: *fliC*, flagellar subunit of H antigen; *stx*, Shiga toxin; *cdt-V*, cytolethal distending toxin V; EHEC*-hlyA*, EHEC hemolysin; α-*hlyA*,

α hemolysin; *espP*, serine protease EspP; *eae*, intimin; *efa1*, EHEC factor for adherence; *lpfA*_O26_, major subunit of long polar fimbriae of EHEC O26; *lpfA*_ O157/OI-141_ and *lpfA*_O157/OI-154_, major subunit of long polar fimbriae of EHEC O157 encoded on O island OI 154 and OI 141, respectively; *iha*, iron-regulated gene A homologue adhesin; *terE*, marker for tellurite resistence-encoding cluster; *ureD*, marker for *ure* cluster encoding urease production; *irp2* and *fyuA*, markers for the high pathogenicity island (HPI) encoding iron uptake system.

b Serotypes were determined using conventional and molecular serotyping; the *fliC* genes indicated were present in both motile and non-motile strains of each respective serotype; NSF, non-sorbitol-fermenting; SF, sorbitol-fermenting.

c −, the gene was absent; +, the gene was present (if the gene was not present in all strains of the respective serotype, the numbers of positive strains are indicated in parenthesis).

d one strain lost *stx* gene before subtyping.

e *espP* subtypes are indicated in parentheses.

f *eae* subtypes are indicated in parentheses.

g a truncated *efa1* gene [34] was present in EHEC O157:H7 and one O145:H28 isolate; complete *efa1* was present in all other strains.

Seven of 10 O157 isolates (two NSF and all five SF), six of 16 (37.5%) O26 isolates, and all O111, O55, O172 and Orough isolates were non-motile, making conventional H typing impossible (Table 1). Subtyping of *fliC* genes encoding the flagellar subunit of the H antigens demonstrated that the non-motile O157 and O26 isolates contained *fliC*_H7_ and *fliC*_H11_, respectively, which was also present in motile isolates of these serotypes. The non-motile isolates of serogroups O111, O55 and O172 contained *fliC*_H8,_ *fliC*_H7,_ and *fliC*_H25_, respectively (Table 1), allowing rapid molecular H typing. *fliC*_H25_ was also present in both Orough:NM isolates (Table 1).

None of the 39 patients from whom EHEC strains were isolated had other intestinal bacterial pathogens (*Salmonella* spp., *Shigella* spp., *Y. enterocolitica*, *C. jejuni*) in their stools.

### Seasonal Distribution of EHEC Serotypes

**Figure 1:**
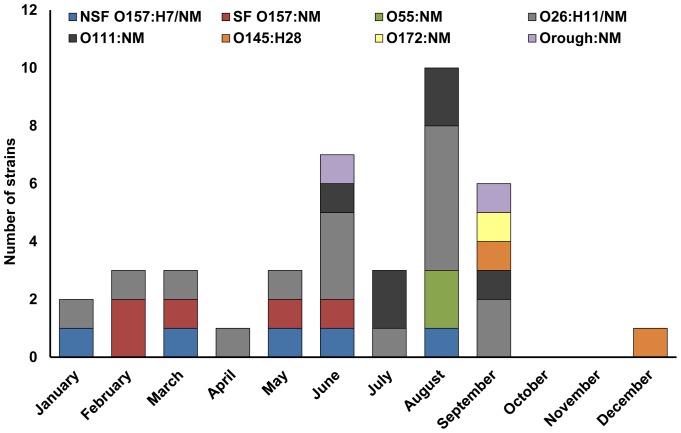
Seasonal distribution of EHEC strains of different serotypes isolated from patients with HUS in the Czech Republic, 1998–2012.

Most EHEC strains (29 of 39; 74.4%) were isolated during the warm period of the year (May to September) (Figure 1). However, no clear seasonality in occurrence of particular serotypes was observed. NSF O157:H7/NM strains were isolated from January through August and SF O157:NM from February to June. O26:H11/NM isolates were almost equally distributed throughout the year (Figure 1).

### *stx* Genotypes

*stx* genes were present in all 39 EHEC isolates upon isolation, but one strain (SF O157:NM) lost its *stx* gene during laboratory subcultures before *stx* subtyping could be performed. Three different *stx* alleles (*stx*_1a_, *stx*_2a_, *stx*_2c_), which through different combinations gave rise to five *stx* genotypes, were identified among the remaining 38 strains (Table 1). The *stx*_2a_ genotype was the most frequent, being present in 27 of 38 (71.1%) strains. The *stx*_2a_ genotype was identified in all strains of serotypes O157:NM (SF), O55:NM, O145:H28, O172:NM, Orough:NM, and in the majority (15 of 16) of O26:H11 strains (Table 1). Only one of five NSF EHEC O157 isolates contained the *stx*_2a_ as the only *stx* gene. The other four NSF O157 strains harbored *stx*_2c_ in combination with either *stx*_1a_ or *stx*_2a_ gene. *stx*_2c_ did not occur in any other serotype (Table 1). *stx*_1a_ as the only *stx* gene was found in three of 38 strains (7.9%) including one of 16 EHEC O26:H11 and two of six O111:NM strains (Table 1).

### Non-*stx* Virulence Genes

Strains of all but one serotype (O55:NM) contained genes encoding non-Stx toxins including cytolethal distending toxin V (Cdt-V) and/or EHEC hemolysin (Table 1). Both *cdt-V* and EHEC-*hlyA* genes were present in four of five SF O157:NM strains and in one NSF O157:H7 strain (Table 1). EHEC-*hlyA*, but not *cdt-V*, was present in all strains of serotypes O26:H11/NM, O145:H28, O172:NM, and Orough:NM, and in four NSF O157:H7/NM and four O111:NM isolates (Table 1). None of the 39 strains contained α-*hlyA* gene encoding α hemolysin (Table 1). All or most strains of serotypes O157:H7/NM (NSF), O26:H11/NM, O145:H28, O172:NM and Orough:NM contained the *espP* gene encoding the plasmid-encoded serine protease EspP [36]. Subtyping of the *espP* genes demonstrated that the O157:H7/NM, O26:H11/NM and O145:H28 strains contain *espP*α, whereas the O172:NM and Orough:NM strains contain *espP*γ; each of these *espP* alleles encodes proteolytically active EspP [36]. *espP* was absent from all strains of serotypes O157:NM (SF), O55:NM, and O111:NM (Table 1).

All 39 EHEC isolates contained the *eae* gene encoding intimin, the major adhesin of EHEC. Four different *eae* subtypes (ß, γ, γ2/θ, ε) were identified, which were associated with particular serotypes. The *eae* γ allele was broadly distributed (being present in all O157, O55, and O145 strains), whereas the other *eae* alleles (ß, γ2/θ, ε) were usually restricted to one serotype (Table 1). In addition to *eae*, genes encoding other established or putative adhesins were found in the EHEC isolates. The *efa1* gene encoding the EHEC factor for adherence (Efa-1) [34] was present in strains of all serotypes (Table 1), but it was truncated in NSF O157:H7/NM and in one of O145:H28 strains as reported previously for *E. coli* O157:H7 [34]. In contrast, loci encoding other adhesins (*lpfA*_O26_, *lpfA*_O157OI-141_, *lpfA*_O157OI-154_, *iha*) were restricted to only some serotypes (Table 1).

### Other Loci

All NSF O157:H7/NM, O26:H11/NM, and O145:H28 strains, and five of six O111:NM strains contained *terE* and *ureD* genes, which were used as markers for the gene clusters encoding tellurite resistance and urease production, respectively. These loci were found in none of the strains of the other serotypes (Table 1). The *irp2* and *fyuA* genes, which are components of the iron-uptake system encoded in the high pathogenicity island (HPI) identified in EHEC by Karch et al. [46] were present only in strains of serotype O26:H11/NM (Table 1).

### Presence of OI 122 in EHEC Isolates

The presence of OI 122 in EHEC strains, and the gene content of this genomic island (i.e. the presence of *pagC*, *nleE*, *nleB*, *sen*, and *efa1* loci) correlate with virulence of EHEC strains [25], [43]. In accordance with their origin from patients with HUS, the EHEC strains characterized in this study contained a complete OI 122 (serotypes O157:H7/NM, O55:NM, O111:NM, and one O145:H28 strain) or an incomplete OI 122 which lacked only *pagC* (serotypes O26:H11, O172:NM, Orough:NM, and one O145:H28 strain) (Table 2).

**Table 2:** Presence of OI 122 among EHEC isolates from HUS patients.

Locus ofOI 122	Serotype (number of strains)							
	O157:H7/NM (NSF)	O157:NM (SF)	O55:NM	O26:H11/NM	O111:NM	O145:H28	O172:NM	Orough:NM
	*fliC*_H7_ (n = 5)	*fliC*_H7_ (n = 5)	*fliC*_H7_ (n = 2)	*fliC*_H11_ (n = 16)	*fliC*_H8_ (n = 6)	*fliC*_H28_ (n = 2)	*fliC*_H25_ (n = 2)	*fliC*_H25_ (n = 1)
*pagC*	+	+	+	–	+	+ (1)^a^	–	–
*nleE*	+	+	+	+	+	+	+	+
*nleB*	+	+	+	+	+	+	+	+
*sen*	+	+	+	+	+	+	+	+
*efa1*	+	+	+	+	+	+	+	+
OI-122^b^	C	C	C	I	C	C (1)I (1)	I	I

a *pagC* was present in one strain.

b C, complete OI 122 (all genes tested present); I, incomplete OI 122 (*pagC* absent).

### Phenotypes

All but one strain (SF O157 which lost *stx* gene) expressed Stx as demonstrated by cytotoxicity of their culture supernatants to Vero cells (Table 1). The Stx type produced by each strain determined using a latex agglutination assay correlated with *stx* genotype. Specifically, strains harboring *stx*_1a_ only produced Stx1a only, those harboring *stx*_2a_ only produced Stx2a only, and those harboring *stx*_1a_+*stx*_2a_ produced both Stx1a and Stx2a (Table 1, Table 3). Stx2c produced by O157:H7 strains with *stx* genotypes *stx*_1a_+*stx*_2c_ or *stx*_2a_+*stx*_2c_ was detected using the Stx2 latex reagent (Table 3). EHEC hemolysin was expressed by all EHEC-*hlyA*-harboring strains of serotypes O157:H7/NM (NSF), O111:NM, O145:H28, and by 15 of 16 EHEC-*hlyA*-containing O26:H11/NM. No EHEC hemolysin production was observed in EHEC-*hlyA*-positive SF EHEC O157:NM strains or in strains of serotypes O172:NM and Orough:NM (Table 3). In accordance with the absence of α-*hlyA* gene (Table 1), none of the 39 EHEC isolates produced a hemolytic phenotype on blood agar (Table 3) Tellurite resistance was expressed in all strains of serotypes O157:H7/NM (NSF), O26:H11/NM, O111:NM, and O145:H28 which contained the *terE* gene, as demonstrated by their ability to grow on CT-SMAC. In contrast, none of the above strains, which also contained *ureD*, produced urease (Table 3). Sorbitol was utilized by all strains except for NSF O157:H7/NM, O172:NM, and Orough:NM as demonstrated by the appearance of their colonies on SMAC and using API 20E test. Rhamnose was utilized by all NSF O157:H7/NM, O111:NM, and O145:H28 strains, but not by strains of the other serotypes (Table 3). All strains except those belonging to serotypes O111:NM, O172:NM and Orough:NM produced lysine decarboxylase (Table 3). All strains but NSF O157:H7/NM produced ß-D-glucuronidase (Table 3).

**Table 3:** Phenotypes of EHEC strains isolated from patients with HUS in the Czech Republic.

Phenotype^a^	Serotype (number of strains)							
	O157:H7/NM (NSF)	O157:NM (SF)	O55:NM	O26:H11/NM	O111:NM	O145:H28	O172:NM	Orough:NM
	*fliC*_H7_ (n = 5)	*fliC*_H7_ (n = 5)	*fliC*_H7_ (n = 2)	*fliC*_H11_ (n = 16)	*fliC*_H8_ (n = 6)	*fliC*_H28_ (n = 2)	*fliC*_H25_ (n = 1)	*fliC*_H25_ (n = 2)
Vero cell titer^b^	32–128	16–512	32–64	16–128	64–2048	256–1024	128	512
Stx 1^c^	–d	–	–	+ (1)^d^	+ (2)	–	–	–
Stx 2^c^	+ (3)	+ (4)	+	+ (15)	–	+	+	+
Stx1+Stx2^c^	+ (2)	–	–	–	+ (4)	–	–	–
EHEC–Hly	+	–	–	+ (15)^e^	+ (4)	+	–e	–e
α–Hly	–	–	–	–	–	–	–	–
CT–SMAC growth	+	–	–	+	+ (5)	+	–	–
Urease	–	–	–	–	–	–	–	–
SMAC	–	+	+	+	+	+	–	–
SOR	–	+	+	+	+	+	–	–
RHA	+	–	–	–	+	+	–	–
LDC	+	+	+	+	–	+	–	–
GLR	–	+	+	+	+	+	+	+

a EHEC-Hly, EHEC hemolysin production; α-Hly, α hemolysin production; growth on CT-SMAC, indicator of tellurite resistance; urease, urease production; SMAC, utilization of sorbitol on sorbitol MacConkey agar; SOR, utilization of sorbitol (API 20E); RHA, utilization of rhamnose (API 20E); LDC, production of lysine decarboxylase; GLR, production of ß-D-glucuronidase.

b The highest dilution of culture supernatant which caused cytotoxicity in 50% Vero cells after 3 days.

c Production of Stx1 and Stx2 tested using the VTEC - RPLA kit.

d −, the phenotype was absent; +, the phenotype was present (the numbers in parentheses indicated numbers of positive strains in the case that not all strains expressed the respective phenotype).

e one O26:H11 and O172:NM and Orough:NM strains did not express EHEC-*hlyA* gene.

### Antimicrobial Susceptibility

Nine of 39 EHEC isolates including five of 16 O26:H11/NM strains, and four of six O111:NM strains were resistant to ampicillin. One of these O26 isolates was also resistant to trimethoprim/sulfamethoxazole. One additional isolate (O145:H28) was resistant to chloramphenicol. All the other 29 strains including all 10 O157 isolates were susceptible to all 12 antimicrobials tested (ampicillin, cefotaxime, ceftazidime, gentamicin, trimethoprim/sulfamethoxazole, ciprofloxacin, amikacin, meropenem, piperacillin/tazobactam, tigecycline, chloramphenicol, and nitrofurantoin).

### Phylogeny of EHEC Associated with HUS

**Figure 2:**
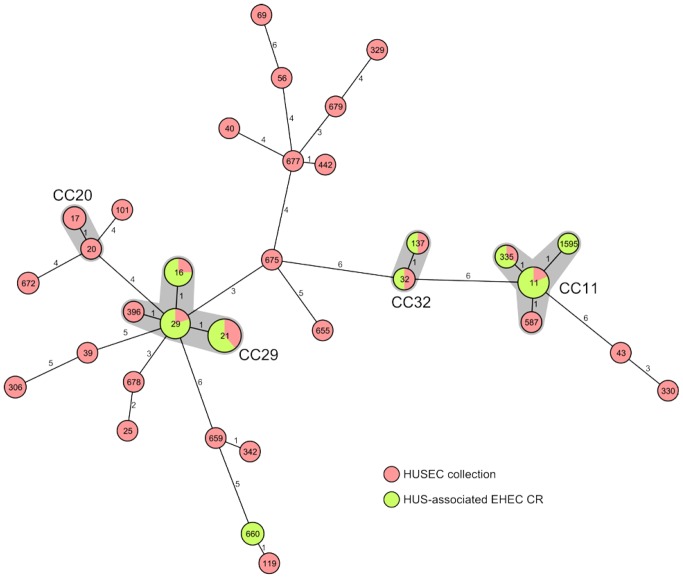
Phylogeny of EHEC associated with HUS in the Czech Republic. Minimum-spanning tree illustrating the clonal relationship between HUS-associated EHEC from the Czech Republic (green) and the HUSEC collection [3] (red) based on MLST allelic profiles. Each MLST sequence type (ST) is represented by a node named with its ST. The size of the node is proportional to the number of isolates reported in this study sharing the same ST. The number on the connecting lines indicates the number of alleles that were different between the two connected nodes. In addition, for the major serogroups (e.g. O157, O26) the STs and their corresponding clonal complexes (CC) were given and shaded in grey.

MLST analysis of the 39 EHEC isolates resulted in nine different STs. Whereas eight of the nine STs clustered into three CCs (CC11, CC29, CC32), ST660 formed a separate clone not clustering into any known CC (Table 4). All EHEC O157:H7/NM (NSF) and O157:NM (SF) belonged, with a single exception (ST1595, which is a single locus variant (slv) of ST11) to ST11 (CC11). Both O55:NM strains (ST335) grouped as a slv of ST11 into the same CC (CC11) as EHEC O157:H7/NM. EHEC O26:H11/NM were equally distributed among two different STs, ST21 and ST29, which clustered together into CC29. The ST29 is composed of strains belonging to the new, highly virulent EHEC O26 clone, which is widespread in Europe [7]. All six strains of serotype O111:NM belonged to ST16 (a slv of ST29) and grouped to CC29 together with EHEC O26 (Table 4). Both O145:H28 strains clustered into CC32 and belonged to ST32 and ST137, respectively, which are slvs. The O172:NM strain and both Orough:NM strains belonged to ST660, suggesting that they have a similar genomic background. Accordingly, a PCR analysis for the presence of the O172 biosynthetic cluster gave a positive result in all three strains demonstrating that they are all genetically O172. Phylogenetic relationships of the Czech HUS-associated strains, the distribution of strains of different serotypes into CCs and the comparison to the HUSEC collection [3] (www.ehec.org) are shown in Figure 2. Interestingly, this comparison revealed the presence of two STs that were not associated with HUS previously, namely ST660 and ST1595.

**Table 4:** Phylogeny of EHEC isolated from HUS patients in the Czech Republic determined by MLST.

Serotype	Total no.of strains	ST (no. of strains)	CC
O157:H7/NM (NSF)	5	11 (4)1595 (1)	1111
O157:NM (SF)	5	11 (5)	11
O55:NM	2	335 (2)	11
O26:H11/NM	16	21 (8) 29^a^ (8)	2929
O111:NM	6	16 (6)	29
O145:H28	2	32 (1)137 (1)	3232
O172:NM	1	660	n.a.
Orough:NM	2	660 (2)	n.a.

ST, sequence type; CC, clonal complex; n.a., not assigned.

a ST29 strains belong to the new EHEC O26 clone [7].

## Discussion

In this 15-year study we systematically investigated stools of patients with HUS for the evidence of EHEC infection. We demonstrate that approximately 70% of patients with D+ HUS contained EHEC strains in their stool samples. Similar to other European countries, EHEC associated with HUS in the Czech Republic involved strains of serogroup O157 and also several non-O157 serogroups. Notably, SF EHEC O157:NM, which were first identified in Germany [47] and later in other European countries [25], [48]–[51], accounted for 50% of all EHEC O157 strains isolated from HUS patients in the Czech Republic during 1998–2012. All SF O157:NM isolates from the Czech Republic possessed *stx*_2a_ gene, similar to such strains from Germany, but not *stx*_1a_ which was identified in SF O157:NM isolated in Norway [50]. The most prevalent EHEC serotype associated with HUS in the Czech Republic is O26:H11/NM, a situation similar to that reported from Italy [26]. Our ability to isolate EHEC strains from all stool samples that tested positive for *stx* genes in the initial PCR screening demonstrates that the EHEC isolation procedure used in this study enables to identify reliably both O157 and non-O157 EHEC strains. In accordance with other studies [3], [37], [49], the large percentage of the Czech EHEC isolates were non-motile. This suggests that non-motility might either be an inherent characteristic of EHEC, in particular of some serogroups, or that such strains rapidly lose their motility *in vitro*, after they have left the host gastrointestinal tract. This observation underlines the importance of the *fliC* genotyping as an easy, rapid and reliable procedure for molecular H typing of EHEC isolates.

All EHEC isolated from HUS patients in the Czech Republic were *eae*-positive as are also most HUS-associated EHEC in other studies [5], [8], [15], [17]. It has been shown in different studies that the rate of *eae*-negative strains among HUS EHEC isolates is low [3], [17], [28]. Mellmann et al. [3] reported that only 16 from 524 (3.1%) EHEC isolates from HUS patients were *eae*-negative. Among them was *E. coli* O104:H4 strain (HUSEC41) [3], which is closely related to the *E. coli* O104:H4 strain that caused the largest ever recorded outbreak of HUS in Germany in 2011 [52], [53], with many secondary cases having occurred worldwide including the Czech Republic [54]. The O104:H4 outbreak strain isolated in 2011 from an American tourist with diarrhea who traveled to Prague from North Germany [54] is the only *eae*-negative EHEC isolated in this country from humans until now. Because *stx*-positive/*eae*-negative strains would have been detected using our PCR screening system, we assume that the absence of *eae*-negative strains among EHEC isolated from HUS patients in the Czech Republic in this study is due to low number of isolates resulting, in turn, from a low number of HUS cases that occur in this country (4–5 per year).

SF EHEC O157:NM caused several HUS outbreaks throughout Europe, the largest of which involved Germany [55], [56], Scotland [57] and Norway [58]. Such strains differ from O157:H7 phenotypically, in particular by their ability to ferment sorbitol and produce β-D-glucuronidase, susceptibility to tellurite, lack of EHEC hemolysin expression and non-motility [41], [47], and by expression of non-Stx toxins that may contribute to the pathogenesis of EHEC-mediated diseases. Specifically, Cdt-V, which causes irreversible injury to microvascular endothelial cells [59], the major targets affected during HUS, is produced by the majority of SF EHEC O157:NM strains [35], but only by a small subset of EHEC O157:H7, which belong to particular phage types [59]. Accordingly, all SF EHEC O157:NM and one NSF O157:H7 strain analyzed in this study harbored the loci encoding Cdt-V (Table 1). EHEC hemolysin, another toxin with a potential endothelium-injuring capacity [60], was regularly expressed by EHEC O157:H7, but not by SF O157:NM strains analyzed in this study (Table 3). Several studies suggest that infections with SF EHEC O157:NM more often progress to HUS [56]–[58] than those with NSF O157 [61] and that patients infected with SF O157:NM have a higher risk of death [56]–[58] than those infected with EHEC O157:H7 [61]. In both German large outbreaks caused by SF EHEC O157:NM strains the case-fatality ratio was 11% [55], [56] compared to <1% reported for outbreaks caused by NSF EHEC O157:H7 [61]. In agreement with the high virulence of SF EHEC O157:NM strains, this strain was the cause of death in one patient in our study. The other two fatal cases were associated with infection by EHEC O26:H11, one of which belonged to the new highly virulent clone, which has emerged in Europe [7] and accounted for 50% of all O26 EHEC O26 isolated in this study.

EHEC O157:H7 have evolved from an *E. coli* O55:H7 ancestor possessing the locus of enterocyte effacement (LEE) by acquisition of Stx-encoding bacteriophages, virulence plasmid and transition of somatic antigen from O55 to O157 [62]–[64]. Leopold et al. [63] and others [65], [66] provided evidence of limited diversity in SF O157:NM, much unlike the large biodiversity of EHEC O157:H7. We show that the NSF and SF O157 isolates described here all belong to the same clonal complex (CC11); multilocus variable number tandem repeat analysis (MLVA) and single nucleotide polymorphism (SNP) analysis of the Czech strains is underway to more extensively compare the phylogenetic relationships of these strains and to compare them with strains from other countries [63], [67], [68].

Tellurite resistance is a diagnostically important feature enabling isolation of EHEC strains from CT-SMAC where normal intestinal flora are suppressed. In our study tellurite resistance occurred in all NSF EHEC O157, all O26 and both O145 isolates and in five of six O111 strains. All these strains also contained the *ure* cluster encoding urease production but none of them produced urease, in accordance with observations that *ure* genes are usually not expressed in EHEC strains [42]. The absence of both *ter* and *ure* loci in one O111:NM strain (Table 1) suggest that the strain have lost these loci, as have been previously reported for EHEC O157:H7 and attributed to deletions within OIs 43 and 48 that harbor these loci [69].

Antimicrobial susceptibility testing demonstrated resistance to one (9 isolates) or two (1 isolate) antimicrobials in 25.6% of the Czech EHEC strains studied. The resistance was associated with non-O157 EHEC serogroups. This situation is similar to that in Finland and Belgium where antimicrobial resistance was reported in 21.4% and 44.7% of EHEC patientś isolates, respectively, and it was more frequent in non-O157 than in O157 strains [70], [71]. As in our study, none of the isolates from these countries was resistant to meropenem or imipenem, ciprofloxacin and amikacin. In contrast to our findings, multidrug resistance occurred in 24.1% of the Belgian [71] and in 7.1% of the Finnish EHEC isolates [70], as well as in an EHEC O145:H^-^ strain that caused a multistate outbreak of diarrhea and HUS in the United States [72]. The extended-spectrum β-lactamase (ESBL) phenotype, which was identified in an EHEC O26 human isolate in another Belgian study [73], and which is one of the typical features of the EHEC O104:H4 strain that caused the large 2011 outbreak in Germany [53], [74] was not tested in the Czech EHEC HUS isolates in this study. Compared to EHEC, *E. coli* strains isolated from urine of patients with urinary tract infections or from blood cultures of patients with sepsis are more often multiresistant [75] or express the ESBL phenotype [76], [77].

The spectrum of EHEC serotypes associated with HUS in the Czech Republic raises the question about reservoirs of these pathogens and sources of human infections in this country. Prevalence of EHEC in cattle feces in the Czech Republic was investigated by Alexa et al. [78]. EHEC shedding was observed in 70% to 100% animals in three different diary farms. EHEC isolates belonged to serogroups O26, O103, O157, O128, and O54, the former three being isolated from HUS patients in our study. Čížek et al. [79] studied the occurence of EHEC O157 in diary farms in the Czech Republic. EHEC O157 strains harboring *stx*_1_, *stx*_2_, *eae*, and EHEC-*hlyA* genes were detected in four of 192 in-line filters examined. Several additional studies identified various animal species as sources of outbreaks or sporadic cases of EHEC infections in this country. In 1995, four cases of HUS in children caused by EHEC O157:H7 were associated with consumption of unpasteurised milk from a farm goat who shed the causative EHEC O157:H7 strain in its feces [80]. Three years later, SF EHEC O157:NM strains were isolated from two siblings (one with HUS and the other with diarrhea) and an epidemiologically associated cow, and a direct contact with the animal was implicated as a possible infection transmission route [81]. This was the first evidence that cattle can be a reservoir of SF EHEC O157 and a source of human diseases [81]. Altogether, these studies demonstrate that similar to other countries [27], [40], [61], cattle and other domestic animals are reservoires of EHEC in the Czech Republic and can be sources of the infection for humans.

We conclude from our data that EHEC strains including O157:H7/NM and a spectrum of non-O157 serotypes are important causes of pediatric D+ HUS in the Czech Republic. Although the spectrum of EHEC serotypes resembles that found in other European countries, the finding of serotypes O172:NM[*fliC*_H25_] and Orough:NM[*fliC*_H25_], which are not members of the German HUSEC collection [3] (www.ehec.org) indicates the need for creating an European collection of HUS-associated EHEC. This collection would enable complex studies of virulence characteristics, mechanisms of adaptation to the human host and evolution of these pathogens, as well as development of optimized methods for their detection.
